# Endoscopy-assisted pars plana vitrectomy in retinal detachments associated with anterior proliferative vitreoretinopathy and epiciliary membranes

**DOI:** 10.1186/s12886-023-03120-y

**Published:** 2023-09-14

**Authors:** Radwan S. Ajlan, Matthew Pfannenstiel, Yong Kam, Harrison Sciulli

**Affiliations:** Department of Ophthalmology, University of Kansas School of Medicine, 7400 State Line Road, Prairie Village, 66208 Kansas City, KS USA

**Keywords:** Anterior hyaloid membrane, Anterior proliferative vitreoretinopathy, Endoscopy, Pars plana vitrectomy, Epiciliary membrane, Proliferative vitreoretinopathy, Tractional retinal detachment

## Abstract

**Background:**

Proliferative vitreoretinopathy (PVR) is the leading cause of recurrent retinal detachment. Anterior PVR can contribute to recurrent retinal detachment and is often difficult to remove during conventional pars plana vitrectomy. The purpose of this study is to report surgical outcomes of single endoscopy-assisted pars plana vitrectomy (E-PPV) in patients with tractional retinal detachments associated with anterior proliferative vitreoretinopathy and epiciliary membranes.

**Methods:**

Retrospective review of E-PPV between 2017 and 2021 at a tertiary referral center. Inclusion criteria involved adult patients who underwent E-PPV for tractional retinal detachment with anterior PVR and epiciliary membranes. Data collection included patients’ demographics, ophthalmic exam findings, and surgical outcomes. A series of independent sample tests of proportion were conducted using a p-value of 0.05 as the threshold for statistical significance.

**Results:**

Eighteen out of 55 patients who underwent E-PPV met the inclusion criteria. There were six females (33%) and 12 males (p-value = 0.096). Age ranged between 27 and 82 years old (mean age 52.1 ± 17.3 years). Nine patients (50%) had a history of ipsilateral retinal detachment repair. Single E-PPV success rate was 100% after three months, and 94.4% at the latest follow up visit. Recurrent retinal detachment with posterior PVR occurred in one patient four months after surgery. Cataract progressed in 57% (8/14) of phakic patients, with 63% (5/8) undergoing cataract extraction surgery within the first postoperative year.

**Conclusion:**

E-PPV enabled epiciliary membrane and anterior PVR visualization and removal. The single E-PPV success rate remained high at the latest follow up visit. E-PPV enabled the preservation of the phakic lens in all study patients. Larger prospective studies are needed on the role of E-PPV in retina surgeries.

## Background

Proliferative vitreoretinopathy (PVR) is the leading cause of recurrent retinal detachment. PVR is responsible for 75% of all primary surgical failures [1]. Postoperative PVR appears within 30 days of surgery 77% of the time, and within 45 days of surgery 95% of the time [2]. Anterior PVR was found in 79% of patients in the Silicone Study [3]. Another study of recurrent retinal detachment after relaxing retinectomy found PVR causing 92.9% of recurrent detachments, and anterior PVR present in 37.5% of cases [4]. Reported anatomical success rate after PVR detachment surgery ranges between 45–85% [2].

PVR can scaffold the posterior hyaloid for cellular migration [5]. Posterior hyaloid membrane (PHM) removal during retinal detachment repair and thorough vitreous shave lower the risk of posterior PVR formation and re-detachment [2]. Chronic retinal detachment, vitreous hemorrhage, multiple retinal breaks, preoperative PVR and comorbid conditions (e.g., proliferative diabetic retinopathy, uveitis) increase the risk of postoperative PVR [6].

**Fig. 1:**
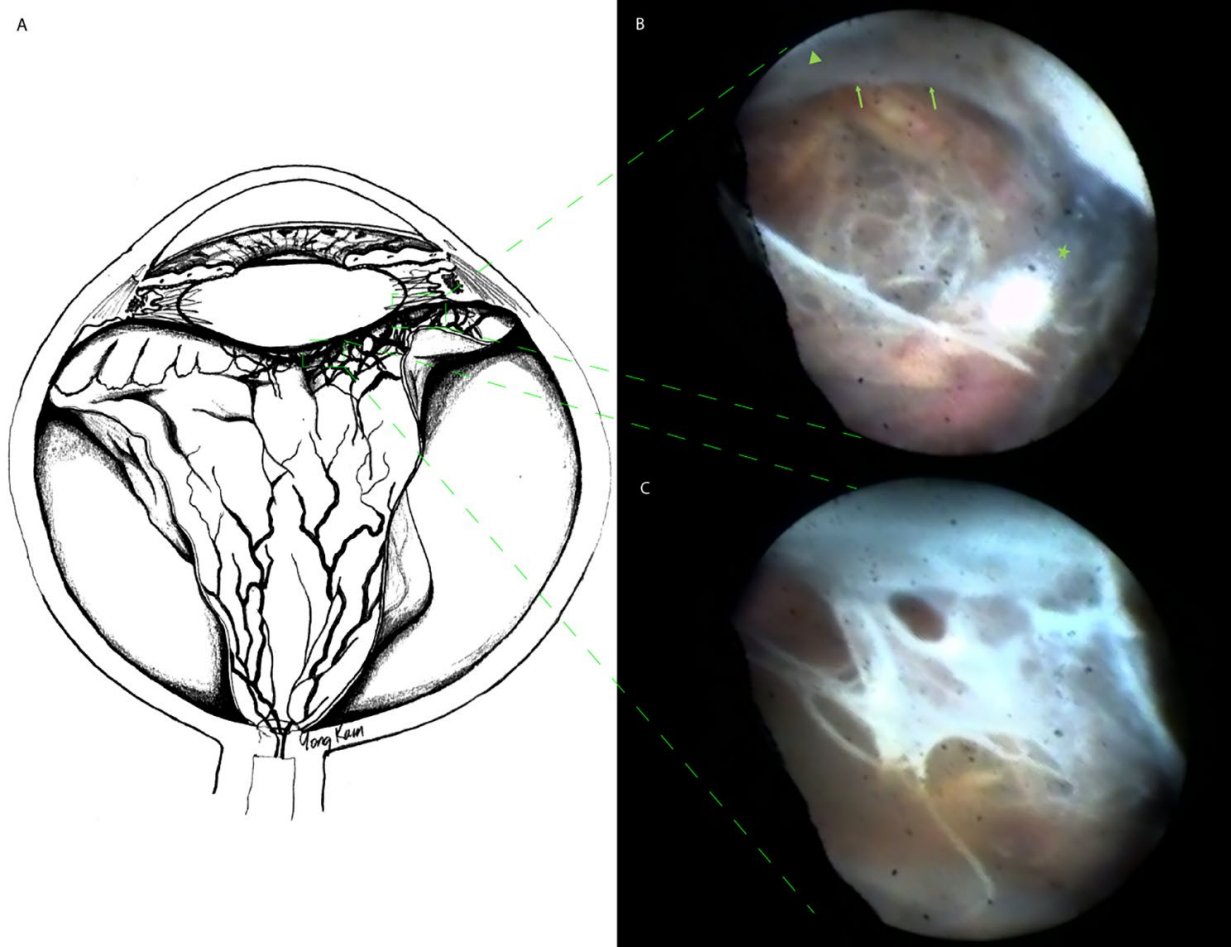
Illustration and endoscopic images are demonstrating anterior proliferative vitreoretinopathy (PVR) in a phakic patient with tractional retinal detachment. An illustration is demonstrating anterior PVR progression from the left to the right side of the diagram with the anterior hyaloid membrane (AHM) involvement **(A).** A surgical endoscopic view is demonstrating the anterior PVR scaffolding over the AHM (green arrows), the phakic lens (green arrowhead), and the vitrector cutter tip (green star). An endoscope probe image artifact is observed on the left side of the surgical view during surgery **(B).** A surgical endoscopic view demonstrating anterior PVR that extends from the retina inferiorly to the AHM and lens capsule superiorly. The same endoscopic probe image artifact is observed on the left side **(C)**

Anterior PVR and fibrovascular proliferation can scaffold anterior vitreous similar to posterior vitreous [7–10]. PVR applies traction on the retina in three-dimensions (i.e., in x, y, and z axes) causing retinal detachment, retinal folds, and ciliary body detachment (Fig. 1A). Anterior PVR removal during pars plana vitrectomy (PPV) frequently requires scleral depression and lensectomy to aid visualization and anterior hyaloid removal. Epiciliary membranes causing tractional retinal detachment or ciliary body detachment cannot be easily visualized using the surgical microscope. Moreover, epiciliary membranes are nearly impossible to visualize through cortical cataracts or dense capsular opacities.

Endoscopy-assisted PPV (E-PPV) improves surgical visualization and enables unique surgical maneuvers. In 2014, Boscher et al. reported thorough eradication of the anterior vitreous to prevent anterior PVR using earlier endoscopy systems (E1 and E2; Endooptiks) [10]. In 2016, Lee et al. found E-PPV useful in removing anterior PVR from the ciliary body to treat chronic hypotony after retinal detachment repair in a minority of cases [11]. Our study reports the outcome of E-PPV in treating retinal detachments with anterior PVR and epiciliary membranes using modern technology.

## Methods

This is a retrospective cohort study of patients who underwent E-PPV for TRD with anterior PVR and epiciliary membrane between December 2017 and August 2021. Data collection included best corrected visual acuity (VA), intraocular pressure (IOP), age, gender, lens status, indication for surgery, postoperative outcome, and complications. The primary outcome is anterior PVR recurrence and anatomical success. All patients were counseled about risks, benefits, alternatives, and informed consent was obtained prior to surgery. Surgeries were performed using a 23-gauge (G) vitrectomy system (Constellation vitrectomy system, Alcon, USA), and the vitreous base was shaved to the safest extent possible using endoscopy to aid visualization. A straight 23G ophthalmic endoscope probe with 10,000-pixel resolution and mounted laser was utilized through the valved 23G cannulas of the vitrectomy system. The E2, Endo Optiks, (Beaver-Visitec International Inc.) with medical grade 17-inch monitor (Beaver-Visitec International Inc), was used for real time endoscopic visualization of the target tissue.

Anterior PVR was removed using a single-handed approach of delamination and segmentation to the safest extent possible utilizing a 23G vitrector handpiece, 23G Grieshaber MaxGrip Forceps, and 23G curved scissor under endoscopic visualization. Epiciliary membranes were removed using a single-handed approach to peel membranes off the ciliary processes using 23G Grieshaber MaxGrip Forceps, 23G curved scissors, and the 23G vitrector handpiece under endoscopic visualization. A peripheral retinectomy was performed when intraretinal PVR was present and preventing retinal tissue repositioning.

The study cohort’s demographics, clinical characteristics, and outcomes were compared. Statistical analysis was performed using (RStudio version 1.1.453). Categorical variables were described using proportions. Continuous variables were described as means ± standard deviations. VA conversion from Snellen chart measurement to LogMAR was calculated using the following formula: [logMAR VA = -log (decimal acuity)] [12]. The values 2.6, 2.7, and 2.8 were used as the LogMAR equivalents for counting fingers, hand motion, and light perception, respectively [13].  A series of independent sample tests of proportion were conducted. A p-value of 0.05 was used as the threshold value for statistical significance, and a confidence interval of 95% was chosen. Microsoft Office Excel 2016 was used for data collection.

## Results

Eighteen out of the 55 patients who underwent E-PPV from December 2017 to August 2021 met the inclusion criteria of undergoing endoscopic TRD repair with preoperative or intraoperative findings of anterior PVR and epiciliary membranes. There was no statistically significant difference in the proportion of females (33.3%, 6/18) versus males (66.7%, 12/18) (p-value = 0.096) in the study. Age ranged between 27 and 82 years old (mean age = 52.1 ± 17.3 years). Preoperative VA ranged from 20/25 to LP (mean logMAR VA 1.66 ± 1.09) and preoperative IOP ranged from three to 24 mmHg (mean IOP = 11.8 ± 5.37 mmHg). Baseline lens status varied with 14 phakic patients (78%), three pseudophakic patients (16.7%), and one aphakic patients (5.6%). There was a statistically significant higher proportion of phakic patients at baseline compared to pseudophakic or aphakic patients (p-value < 0.00001). There was no significant difference between preoperative retinal surgery status as nine patients (50%) had a history of ipsilateral retinal detachment with prior surgical repair while nine (50%) patients were retinal surgery naïve (p-value = 1). Anterior PVR was visualized during preoperative exam except when vitreous hemorrhage was blocking the view (22.2%, 4/18). B-scan ultrasonography was performed preoperatively on all patients who had anterior PVR documented before surgery. Ultrasound biomicroscopy (UBM) was performed in three patients with preoperative hypotony (16.7%), those patients had epiciliary membranes detected before surgery. The other 15 patients (83.3%) had epiciliary membranes detected during surgery. The patient demographics and indications for surgery are summarized in Table 1.

During surgical repair, all patients 100% (18/18) had successful removal of anterior PVR and epiciliary membranes, 39% (7/18) of patients underwent peripheral retinectomy, and one pseudophakic patient underwent manual posterior capsulotomy (5.6%). The intraocular tamponade used at the end of surgery was silicone oil (5000cs) in 83% (15/18) and gas in 17% (3/18) with a statistically significant higher proportion of patients undergoing silicone oil tamponade than gas tamponade (p-value = 0.00025). After surgery, 33% (5/15) of patients had silicone oil removed within one year (at an average of 4 ± 0.89 months after surgery).

All patients were examined after three months, and 78% (14/18) attended a postoperative exam at one-year. At the final visit, postoperative VA ranged from 20/20 to LP (mean LogMAR 1.76 ± 1.16) and postoperative IOP ranged from two to 22 mmHg (12.6 ± 5.46 mmHg). Cataract progressed in eight out of 14 phakic patients (57%). Five of the eight patients with cataract progression (63%) had cataract extraction surgery within one year. There were no posterior lens capsule defects noted during cataract extraction surgery (Table 1).

One patient (5.6%) re-detached within one year of surgery. He initially presented with a branch retinal vein occlusion (BRVO) and chronic anterior TRD. He underwent E-PPV with silicone oil tamponade. Four months later, his retina re-detached because of recurrent posterior PVR along the inferior-temporal arcade, the foveal center was attached under silicone oil. Further surgical repair was offered but the patient deferred additional intervention. He was examined after one year and his fovea remained attached under oil (Table 1).

**Table 1:** Demographics and Outcomes

Age	Gender	Indication for Surgery	Pre op VA	Preop IOP	Preop Lens Status	Retinectomy	Vitreous Tamponade	Final postop VA	Final postop IOP	Re-detachment	One year follow up
29	Male	TRD, PDR	20/50	14	Cataract	No	Silicone Oil	20/20	13	No	Yes
43	Female	TRD, PDR	LP	12	Cataract	No	Silicone Oil	LP	13	No	No
67	Male	TRD, VH, PDR	20/40	14	Cataract	No	Sulfur Hexafluoride (SF6 gas) at 20% concentration	20/40	17	No	Yes
37	Male	TRD	20/25	16	Clear	No	Silicone Oil	20/30	12	No	Yes
38	Male	TRD, PDR	HM	4	Clear	Yes (360 degree)	Silicone Oil	LP	12	No	Yes
49	Female	TRD, VH, PDR	20/100	13	Cataract	No	Silicone Oil	20/600	20	No	Yes
72	Male	Re-TRD	CF	17	Aphakic	Yes (180 degree)	Silicone Oil	HM	21	No	Yes
49	Female	TRD, PDR	LP	14	Cataract	No	Silicone Oil	HM	14	No	Yes
32	Male	TRD, VH, PDR	20/50	14	Clear	No	Silicone Oil	20/30	15	No	Yes
27	Male	TRD	20/400	11	Clear	No	Silicone Oil	20/200	11	No	No
51	Female	TRD, PDR	20/40	14	Clear	Yes (150 degree)	Silicone Oil	CF	12	No	No
67	Male	TRD	20/1000	24	Cataract	Yes (180 degree)	Silicone Oil	20/400	8	No	Yes
80	Female	TRD	HM	3	PCIOL	Yes (210 degree)	Silicone Oil	HM	5	No	Yes
82	Female	TRD	20/1250	3	PCIOL	No	Octafluoropropane (C3F8 gas) at 15% concentration	HM	2	No	Yes
35	Male	TRD, VH, PDR	20/200	15	Clear	No	Sulfur Hexafluoride (SF6 gas) at 20% concentration	20/25	15	No	No
62	Male	TRD	HM	9	Clear	Yes (300 degree)	Silicone Oil	HM	22	Yes	Yes
56	Male	TRD	LP	10	Cataract	Yes (180 degree)	Silicone Oil	HM	10	No	Yes
62	Male	TRD, choroidal hemorrhage post ruptured globe	LP	6	Aphakic	No	Silicone Oil	HM	5	No	Yes

## Disscusion

E-PPV facilitated anterior PVR removal and epiciliary membrane peel in patients with anterior TRD. Single E-PPV anatomical success rate was 100% after 3 months, and 94% at the final follow up visit. None of the patients required lensectomy or lens exchange during E-PPV. Cataract progressed in 57% of phakic patients after one year with the majority of patients undergoing cataract extraction within the year. No lens capsule injury was detected in patient who underwent cataract extraction.

Anterior PVR removal can increase the success rate of retinal repair surgery. In 2006, Quiram et al. reported improved reattachment rates when anterior vitreous base dissection and lensectomy were performed at the time of retinectomy in patients with PVR compared to patients who did not undergo lensectomy (74% vs. 38%, respectively, p-value = 0.011) [14]. Complete retinal attachment rate was 93% with repeat surgery [14].

In 2020, Rezende et al. reported E-PPV was advantageous compared to PPV alone in patients with retinal detachments and PVR [15]. The PPV-only group had an average number of 4.1 surgeries during a mean follow up duration of 31.9 months. The E-PPV group had an average number of 2.7 surgeries during a mean follow up duration of 21.1 months. The final reattachment rate in the PPV-only vs. E-PPV groups was 79% vs. 95%, respectively. Increased final reattachment rate was attributed to the ability to detect previously undetected pathology (e.g., anterior PVR traction) and access anterior and peripheral pathology under endoscopic visualization [15].

In our study, endoscopic visualization facilitated dissection of anterior PVR and removal of epiciliary membranes with anterior hyaloid membrane (AHM) when needed (Fig. 1B C). The AHM along with anterior PVR were dissected off the lens without compromising the lens capsule in all phakic patients (78% of study patients). Retinectomy was performed in 39% (7/18) of patients when intraretinal PVR prevented retinal attachment. None of the patients developed anterior PVR but one patient developed posterior PVR. The final anatomical success rate after single E-PPV was 94% (17/18). Anterior vitreous removal may have contributed to higher surgical success rates.

Epiciliary membranes may cause ciliary body detachments and intractable hypotony [11]. Lensectomy facilitates anterior PVR removal but does not remove cyclitic membranes (Fig. 1C). Peripheral retinectomy separates the retina from cyclitic membranes, but residual cyclitic membranes may subsequently contract causing ciliary body detachment and postoperative hypotony. In our study, all patients had peripheral PVR and epiciliary membranes removed. The majority of patients (78%, 14/18) underwent AHM dissection, the rest were pseudophakic or aphakic. E-PPV provided visualization of peripheral anterior PVR for surgical removal and AHM dissection without disrupting the barrier between the anterior and posterior segments.

An intact anterior-posterior segment barrier provides superior posterior segment tamponade, lowers the risk of gas or silicone oil migration into the anterior chamber, and spares patients the need for inferior iridotomy with silicone oil [16–18]. Performing vitrectomy with mechanical posterior capsulotomy in pseudophakic eyes removes the central portion of the anterior hyaloid but not the peripheral AHM near lens zonules nor anterior PVR overlying the ciliary bodies. Lensectomy offers more complete removal of the AHM and can maintain separation of the anterior and posterior segments when the anterior lens capsule is preserved. Following lensectomy, intraocular lens implants can be placed in the sulcus or in the anterior chamber. In our study, E-PPV provided additional surgical view and facilitated peripheral anterior PVR removal without lensectomy or lens exchange. This allows for lens implantation in the capsular bag when cataract removal is needed.

The reported rate of cataract formation after PPV is up to 100% within the first two years [19]. PPV increases oxygen tension and circulation in the vitreous cavity of vitrectomized eyes [20]. Do et al. have demonstrated that increased intraocular oxygen tension post PPV may play a role in the accelerated rate of postoperative cataract formation [21]. AHM peel exposes the posterior lens capsule to the vitreous fluid, which may increase intraocular oxygen tension on the phakic lens. These speculations remain to be investigated. In our study, eight out of the 14 phakic patients (57%) had cataract progression. Five of the eight patients with progressed cataracts underwent cataract extraction surgery during the first postoperative year. None of the cataract patients had posterior lens capsule defect reported during cataract extraction.

Endoscopic coaxial illumination facilitates vitreous and membrane visualization. This improved visualization reduces the risk of iatrogenic injury to adjacent intraocular structures, such as the posterior capsule, lens zonule, or ciliary body [22, 23]. A 23G endoscope probe with 10,000-pixel image resolution was used for all patients in this study. Currently in the United States, this probe is the smallest available ophthalmic endoscope approved by the Food and Drug Administration [24]. Globally, ophthalmic endoscope probes are available in smaller gauges as 25G and 27G [25]. 25G endoscopes can provide up to 10,000-pixel image resolution [25].

Repeat use of the same endoscope probe is associated with decreased illumination and image quality, which can result from damage during sterilization. Chandelier illumination can be used during E-PPV to improve illumination. Another option is to use a large monitor screen to facilitate endoscopic tissue visualization with reduced illumination [26].

This study has several strengths, a key example is the one-year postoperative follow up for 78% of patients. While 95% of postoperative PVR occurs during the first 45 days, our study’s extended follow up allowed for the discovery of PVR formation at four months. Another strength is tracking cataract formation in phakic patients after anterior PVR, anterior hyaloid, and cyclitic membrane removal. Cataract progressed in eight out of 14 phakic patients (57%) and five of the eight patients with cataract progression (63%) underwent cataract extraction surgery within one year. The study had several limitations because of its retrospective nature. For example, there was no control group and no standardized randomization of patients in this study. Additionally, the sample size (18 patients) was limited by the retrospective nature of the study and infrequent pathology. Patient age (mean 52.29 ± 17.33 years) may also be a limitation as younger patients have more risk of PVR formation compared to older patients.

## Conclusions

In conclusion, the single E-PPV success rate for this study’s patients was 100% after 3 months, and 94% at the latest follow up visit. E-PPV improved anterior PVR visualization and facilitated epiciliary membrane removal. Additionally, E-PPV allowed for phakic lens preservation providing the option for future conventional cataract extraction. Our study provides support for the growing evidence of E-PPV benefits. Larger prospective studies are needed to better understand the role of E-PPV in retina surgeries.

## Data Availability

All data generated or analyzed during this study are included in this published article.

## Abbreviations

PVR: proliferative vitreoretinopathy
PHM: posterior hyaloid membrane
AHM: anterior hyaloid membrane
BSS: balanced salt solution
E-AHM: endoscopic anterior hyaloid membrane
KUMC: University of Kansas Medical Center
E-PPV: endoscopy-assisted pars plana vitrectomy
VA: visual acuity
IOP: intraocular pressure
G: gauge
SCH: suprachoroidal hemorrhage
TRD: tractional retinal detachment
VH: Vitreous hemorrhage
